# NF-κB Signaling Regulates Epstein–Barr Virus *Bam*HI-Q-Driven *EBNA1* Expression

**DOI:** 10.3390/cancers10040119

**Published:** 2018-04-16

**Authors:** Rob J. A. Verhoeven, Shuang Tong, Jingfeng Zong, Yixin Chen, Sai-Wah Tsao, Jianji Pan, Honglin Chen

**Affiliations:** 1 Department of Microbiology and State Key Laboratory for Emerging Infectious Diseases, The University of Hong Kong, Hong Kong, China; rjav@connect.hku.hk (R.J.A.V.); clare729@connect.hku.hk (S.T.); 2 Department of Radiation Oncology, Fujian Provincial Cancer Hospital, Provincial Clinical College of Fujian Medical University and Fujian Provincial Key Laboratory of Translational Cancer Medicine, Fuzhou 350014, China; zongjingfeng@126.com (J.Z.); panjianji@126.com (J.P.); 3 National Institute of Diagnostics and Vaccine Development in Infectious Diseases, School of Life Sciences, Xiamen University, Xiamen 361005, China; yxchen2008@xmu.edu.cn; 4 School of Biomedical Sciences, Li Ka Shing Faculty of Medicine, The University of Hong Kong, Hong Kong, China; gswtsao@hku.hk

**Keywords:** Epstein–Barr virus, NF-κB, EBNA1

## Abstract

Epstein–Barr virus (EBV) nuclear antigen 1 (EBNA1) is one of the few viral proteins expressed by EBV in nasopharyngeal carcinoma (NPC), most likely because of its essential role in maintaining the viral genome in EBV-infected cells. In NPC, EBNA1 expression is driven by the *Bam*HI-Q promoter (Qp), which is regulated by both cellular and viral factors. We previously determined that the expression of another group of EBV transcripts, *Bam*HI-A rightward transcripts (BARTs), is associated with constitutively activated nuclear factor-κB (NF-κB) signaling in NPC cells. Here, we show that, like the EBV BART promoter, the EBV Qp also responds to NF-κB signaling. NF-κB p65, but not p50, can activate Qp in vitro, and NF-κB signaling regulates *Qp-EBNA1* expression in NPC cells, as well as in other EBV-infected epithelial cells. The introduction of mutations in the putative NF-κB site reduced Qp activation by the NF-κB p65 subunit. Binding of p65 to Qp was shown by chromatin immunoprecipitation (ChIP) analysis, while electrophoretic mobility shift assays (EMSAs) demonstrated that p50 can also bind to Qp. Inhibition of NF-κB signaling by the IκB kinase inhibitor PS-1145 resulted in the downregulation of *Qp-EBNA1* expression in C666-1 NPC cells. Since EBNA1 has been reported to block p65 activation by inhibiting IKKα/β through an unknown mechanism, we suggest that, in NPC, NF-κB signaling and EBNA1 may form a regulatory loop which supports EBV latent gene expression, while also limiting NF-κB activity. These findings emphasize the role of NF-κB signaling in the regulation of EBV latency in EBV-associated tumors.

## 1. Introduction

Epstein–Barr virus (EBV) is a ubiquitous human gammaherpesvirus that is able to establish lifelong latent infection in vivo. Although primary EBV infection is generally without serious symptoms, it may cause infectious mononucleosis in teenagers and has been found to associate with various malignancies of lymphoid and epithelial origin [1]. During EBV latency in vivo, viral gene expression is tightly regulated, with very few essential viral antigens being expressed, while the viral genome is stably maintained as cells divide [2]. Although EBVs in different associated tumors exhibit different latent protein expression patterns, Epstein–Barr nuclear antigen 1 (EBNA1) expression is always present. EBNA1 is a DNA-binding protein which plays an essential role in the replication and maintenance of the episomal EBV genome through binding to the plasmid origin of viral replication (oriP) [3]. EBNA1 also binds to other viral gene promoters and functions as a transcriptional transactivator to regulate the expression of latent membrane protein 1 (LMP1) and other EBNAs [4].

EBNA1 is a latent EBV protein which is expressed in all EBV infections, including the associated malignancies. EBNA1 expression is characteristically mediated by alternative promoters in different forms of EBV latency. Initially, the expression of EBNA1 and other EBNA proteins in EBV-infected B cells is driven by the *Bam*HI-W promoter (Wp), with differential splicing producing various EBNA transcripts [5]. The *Bam*HI-C promoter (Cp) is then activated by EBNA1 and EBNA2 and drives the full spectrum of EBNA expression typical of the latency III program, which is associated with EBV-associated posttransplant lymphoproliferative diseases, EBV-transformed lymphoblastoid cell lines, and infectious mononucleosis. In EBV-associated cancers like Burkitt’s lymphoma and nasopharyngeal carcinoma (NPC), EBNA expression is switched from the Cp to the TATA-less *Bam*HI-Q promoter (Qp), leading to the exclusive expression of EBNA1, but no other EBNAs (EBNA2 and EBNA3 family), defined as latency types I and II, respectively [1,6,7]. Analysis of the EBV genome in Burkitt’s lymphoma and NPC showed that the shutdown of Cp is largely caused by DNA methylation in the Cp promoter region [8,9,10]. Exceptionally, one study has shown that the expression of Qp-initiated EBNA1 transcripts in lymphoblastoid cell lines can be induced by heat treatment, through the binding of heat shock factor 1 (HSF1) to Qp [11].

As Qp-driven EBNA1 latency is exclusively used to maintain EBV infection in Burkitt’s lymphoma, NPC, and EBV-associated gastric carcinoma [6,7], the mechanism for regulation of Qp has been extensively studied [12,13,14]. Two EBNA1 binding sites are located downstream of the RNA initiation site and are negatively regulated by EBNA1, resulting in an autoregulatory loop [15]. However, this repression by EBNA1 can be counteracted by the E2F family of transcription factors, which bind to the DNA at a site between the two binding sites for EBNA1 and interfere with EBNA1 suppression [16]. Different interferon regulatory factors (IRFs) modulate Qp either negatively (IRF2 and 7) or positively (IRF1 and 2) through binding to motifs close to the RNA initiation site [12,13,17]. Interestingly, our previous study identified two signal transducer and activator of transcription (STAT) binding sites, one of which overlaps with the IRF binding site, suggesting that IRFs and STATs may coordinate transcription in a particular way to regulate Qp [14]. This study also presented evidence showing the downregulation of Qp activity by the lytic transactivator Zta, through p53-mediated interference of the Janus kinase (JAK)/STAT pathway [14].

Although the principal function of EBNA1 is to facilitate the replication of the viral episome in EBV-infected cells, a more recent finding showed that EBNA1 can inhibit the canonical nuclear factor-κB (NF-κB) pathway in NPC cells by inhibiting IκB kinases (IKKs) [18]. Considering the autoregulatory loop between NF-κB signaling and LMP 1 and the regulation of *Bam*HI-A rightward transcripts (BARTs) by NF-κB reported by us previously, we hypothesized that the regulatory role of NF-κB may also encompass Qp-driven EBNA1 expression in NPC [19]. This idea was strengthened by the discovery of an NF-κB consensus sequence in the Qp region, just upstream of the previously identified STAT binding sites. In this study, we sought to determine whether NF-κB also plays a role in the regulation of Qp-initiated *EBNA1* (*Qp-EBNA1*) gene expression in EBV-associated NPC.

## 2. Results

### 2.1. NF-κB p65 Upregulates Qp Activity

**Figure 1:**
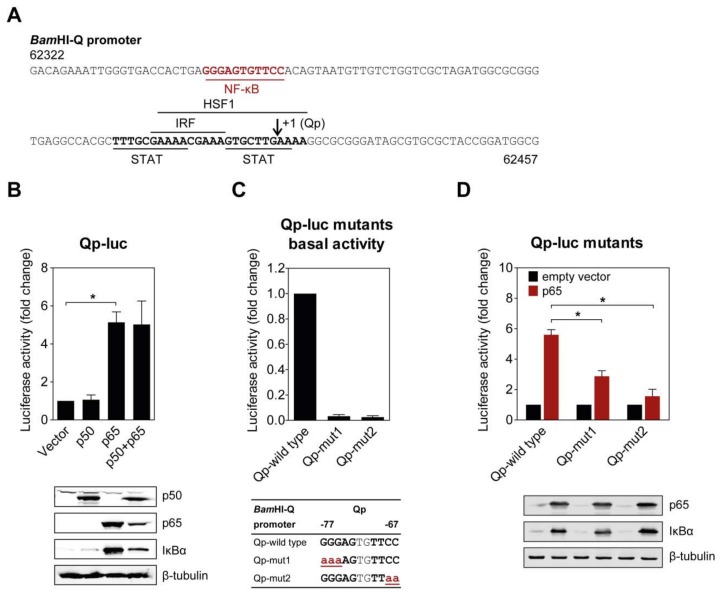
NF-κB p65 activates the *Bam*HI-Q promoter (Qp). (**A**) Sequence of the Qp region showing the locations of previously identified transcription factor binding sites (bold and underlined), the putative NF-κB site (red, bold, and underlined), and the RNA initiation site (arrow). The sequence numbering is based on the Epstein–Barr virus (EBV) B95-8 genome; (**B**) HEK 293T cells were co-transfected with the empty vector or p50 and p65 expression vectors, together or separately, with the Qp reporter; (**C**) The effect of mutations in either the p50 or p65 half-site on the basal expression of the Qp reporter. The mutations are shown below the graph in bold red lowercase font, underlined; (**D**) Effect of the mutations from (**C**) on the activation of Qp by p65. The expression of p50, p65, IκBα, and β-tubulin was detected by immunoblotting using specific antibodies. The luciferase activity is shown as fold-change luciferase activity by normalizing firefly/renilla ratios to the vector control. The averages and standard errors of the mean (SEM) from three independent experiments are shown. Statistical significance was calculated using the two-tailed Student’s *t*-test. * *p* < 0.05.

Several binding motifs in the Qp region, corresponding to IRF, STAT, and HSF1, have been identified in previous studies [11,12,14]. However, these host factors only moderately activate Qp activity, suggesting that other uncharacterized transcriptional factors may also bind Qp. Examination of the Qp promoter revealed that there is a potential binding site for NF-κB located further upstream of the binding sites for other host factors (Figure 1A). To investigate the ability of NF-κB to activate Qp in cells, p50 and p65 expression plasmids were co-transfected either separately or together with the Qp luciferase reporter in human endothelial kidney (HEK) 293T cells. While no apparent activation was induced by p50 alone, Qp promoter activity was significantly increased (approximately 5-fold) when p65 was transfected alone or co-transfected with p50, suggesting a crucial role for p65 in Qp regulation (Figure 1B). Immunoblot analysis showed that the difference between p50 and p65 in Qp activation was not due to a lack of p50 protein expression. Upregulation of IκBα in response to p65 overexpression was observed, confirming high canonical NF-κB activity.

### 2.2. Mutations in the Putative NF-κB Binding Site Attenuate Qp Activation by NF-κB p65

The functional significance of the NF-κB binding site identified in the Qp promoter region was confirmed by site-directed mutagenesis of both NF-κB half sites and examination of promoter activity in a reporter assay. Mutation of either half site led to a complete disappearance of basal Qp activity in the cells (Figure 1C). Both mutated Qp reporters also showed significantly reduced responsiveness to activation by p65 compared to the wild type Qp reporter (Figure 1D), further confirming the involvement of NF-κB in Qp activation. Both NF-κB half sites seemed to be involved in p65-mediated activation of Qp. Taken together, these results demonstrate that the EBV Qp promoter contains an NF-κB binding site which is positively regulated by NF-κB signaling.

### 2.3. Binding of NF-κB to Qp

**Figure 2:**
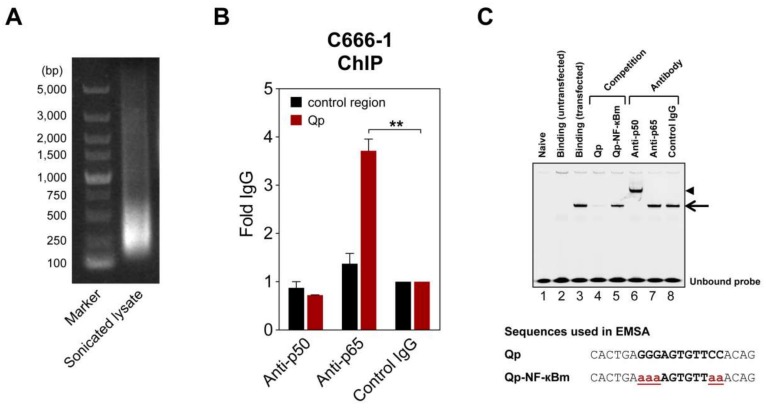
NF-κB can bind Qp. (**A**) Representative image of an agarose gel showing DNA present in a sonicated C666-1 cell lysate used for chromatin immunoprecipitation (ChIP) experiments. (**B**) ChIP of NF-κB p50 and p65 with Qp in C666-1 cells. Results of real-time PCR analysis ChIP assays with antibodies specific for p50 and p65 and a rabbit control IgG are shown. The results are expressed as fold enrichment, where the rabbit control IgG was set to 1. A genomic region ~5 kbp upstream of the IL-8 promoter was used as the negative control region. The means and SEM from three independent experiments are shown, and all samples were analyzed in triplicate. Statistical significance was calculated using the two-tailed Student’s *t*-test. ** *p* < 0.01. (**C**) An IRDye700-labeled DNA probe corresponding to the Qp region, spanning positions -83 to -63 of the B95-8 EBV sequence, was incubated with nuclear extracts from HEK 293T cells transfected with p50 and p65 and subjected to EMSA. Lane 2 shows the binding pattern of the nuclear extract from untransfected cells, while lane 3 shows the binding band associated with transfected cells, indicated by an arrow. Lanes 4 and 5 show the binding of the probe when an excess of a competitor oligonucleotide was added to the binding reaction. Super-shift experiments with antibodies were performed as indicated above the gel; the super-shifted complex is indicated by an arrowhead. Nucleotide sequences of the double-stranded probes and oligonucleotides used in the competition experiments are shown below the gel image. The NF-κB binding site in the Qp promoter is in bold uppercase letters, while the mutated nucleotides are in bold red lowercase letters and underlined. The gel displayed is representative of results obtained in two independent experiments.

To further verify NF-κB regulation of Qp, chromatin immunoprecipitation (ChIP) and electrophoresis mobility shift assay (EMSA) experiments were performed to test whether NF-κB binds to the Qp promoter. In ChIP experiments using lysates from EBV-harboring C666-1 cells, only the p65-specific antibody significantly enriched Qp promoter DNA (>3-fold over control IgG antibody), which is in line with the findings from the reporter assays (Figure 2B). EMSA was performed to confirm the ability of NF-κB to bind the putative NF-κB motif located between positions -77 and -67 upstream of the RNA initiation site of Qp (Figure 1A). An IRDye700-labeled probe covering the putative Qp NF-κB site (-83/-63) was used in EMSA with nuclear extracts prepared from HEK 293T cells transfected with pcDNA3.1-EGFP-p50 and pcDNA3.1-Flag-p65. Real-time PCR analysis ChIP assays with antibodies specific for p50 and p65 showed that the promoter region of Qp was enriched with anti-p65 but not with anti-p50 or control IgG antibodies (Figure 2A,B). While the nuclear extract from untransfected cells did not contain any shifted protein–DNA complexes (Figure 2C, lane 2), the nuclear extracts prepared from cells transfected with pcDNA3.1-EGFP-p50 and pcDNA3.1-Flag-p65 showed a single clear binding band (Figure 2C, lane 3). The specificity of the shifted band was confirmed by using an excess of unlabeled probe, which resulted in an absent band when an unlabeled wild-type oligonucleotide was used as a competitor, but not when a mutated version of the oligonucleotide was used (Figure 2C, lanes 4 and 5). The mutations in the mutated competitor oligonucleotide used in the EMSA experiment corresponded to the introduced mutations in the reporter experiments. The addition of p50-specific antibody to the binding reaction resulted in a super-shift, suggesting that the p50 subunit did bind to the Qp promoter (Figure 2C, lane 6). However, the p65-specific antibody failed to super-shift the specific protein-DNA complex, which, while unexpected in the context of the results presented above, reflects our observations in EMSA experiments investigating p65 binding to EBV BART promoters [20]. We suspect that the p65-specific antibody is not suitable for use in the EMSA super-shift assay. Regardless, the ChIP and EMSA experiments provide evidence that both subunits of NF-κB can bind the Qp promoter, resulting in promoter activation.

### 2.4. NF-κB Activation in EBV-Positive Epithelial Cells Upregulates Qp-EBNA1 Expression

**Figure 3:**
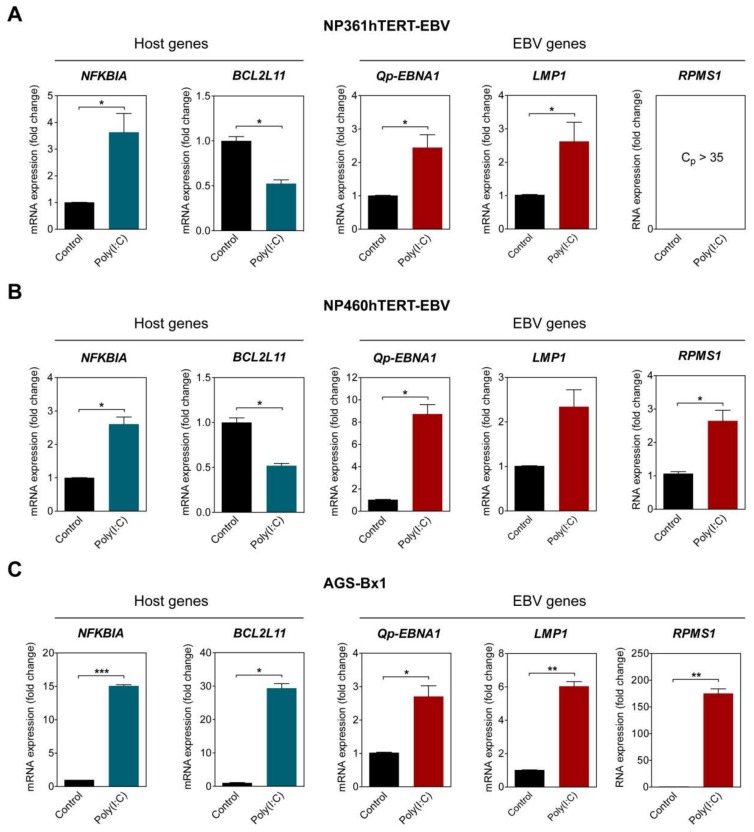
NF-κB activation upregulates *Qp-EBNA1* expression. The nasopharyngeal epithelial (NPE) cell lines NP361hTERT-EBV (**A**) and NP460hTERT-EBV (**B**) and the gastric cell line AGS-BX1 (**C**) were stimulated with poly(I:C) for 16 h. RT-qPCR was performed to analyze changes in the expression of the *NFKBIA*, *BCL2L11, Qp-EBNA1*, *LMP1*, and *RPMS1* genes. Gene expression is shown as fold change mRNA expression relative to that of *GAPDH*. The averages and SEM from at least two independent experiments are shown. Statistical significance was calculated using the two-tailed Student’s *t*-test. * *p* < 0.05, ** *p* < 0.01, *** *p* < 0.001.

To examine if NF-κB activity plays a role in upregulating Qp-initiated *EBNA1* (*Qp-EBNA1*) mRNA expression in EBV-infected cells, three different EBV-infected epithelial cell lines exhibiting latency II were stimulated with poly (I:C). The activation of the NF-κB pathway was confirmed by analyzing the expression of *NFKBIA*, which is controlled by a promoter highly responsive to NF-κB and encodes the protein IκBα, providing autoregulation of NF-κB signaling [21]. The EBV-infected NP460hTERT-EBV and AGS-BX1 cell lines were stimulated with 0.5 µg/mL poly (I:C), while NP361hTERT-EBV cells were stimulated with 1 µg/mL poly (I:C) to achieve a similar effect on *NFKBIA* expression. As expected, treatment with an appropriate amount of poly (I:C) resulted in the upregulation of *NFKBIA* expression in all tested cell lines, indicating activation of the NF-κB pathway (Figure 3A–C). The expression of *BCL2L11*, which encodes the pro-apoptotic protein Bim and has been previously shown to be downregulated by NF-κB activity, was examined as a negative control [22]. The levels of *Qp-EBNA1* were upregulated approximately 8-fold in NP460hTERT-EBV and about 2.5-fold in NP361hTERT-EBV and AGS-BX1 cells (Figure 3A–C). In NPC cells, EBV expresses EBNA1, BARTs, and variable levels of LMP1. Expression of the EBV transcripts *LMP1* and *RPMS1* (BART lncRNA) are also known to be regulated by NF-κB (20). Stimulation with poly (I:C) resulted in a modest upregulation of *LMP1* (2.5- and 6-fold) in NP361hTERT-EBV and AGS-BX1 cells, respectively (Figure 3A,C). For unknown reasons, epithelial cells artificially infected with EBV express very low levels of both BART miRNA and lncRNA, as observed here and in our previous study (Figure 3A) [23]. However, induction of NF-κB activity did upregulate *RPMS1* expression in NP460hTERT-EBV cells (Figure 3B). It seems that AGS-BX1 is more responsive to poly (I:C). Notably, *RPMS1* expression in AGS-BX1 cells was induced nearly 200-fold following poly (I:C) stimulation (Figure 3C). Interestingly, the expression of *BCL2L11* responded differently to poly (I:C) treatment in AGS-BX1, which may be induced by NF-κB independent pathways. Together, these data provide evidence that NF-κB signaling plays a general role in activating the expression of the EBV latency-associated regulators EBNA1, LMP1, and BART-lncRNA in EBV-infected epithelial cells.

### 2.5. Nuclear Localization of NF-κB p65 in an EBV-Positive NPC Cell Line 

**Figure 4:**
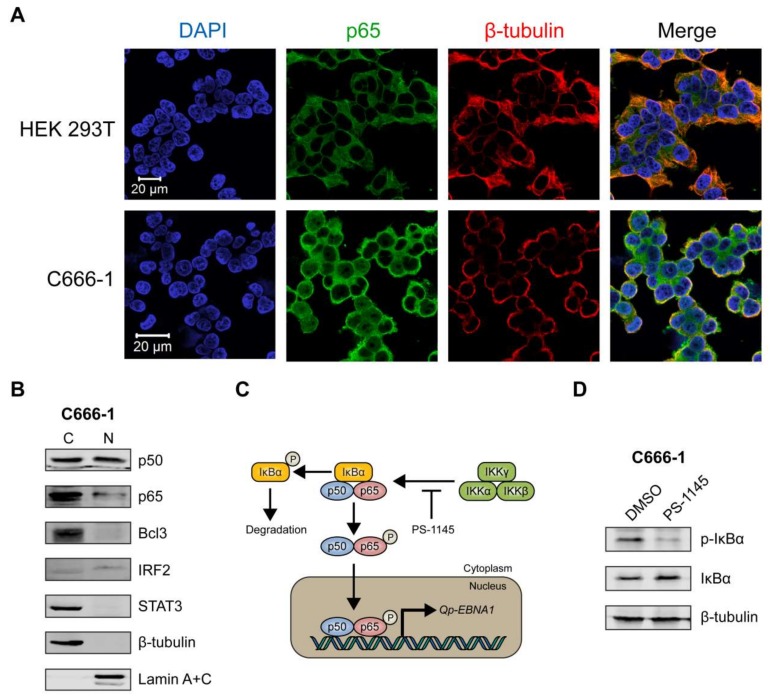

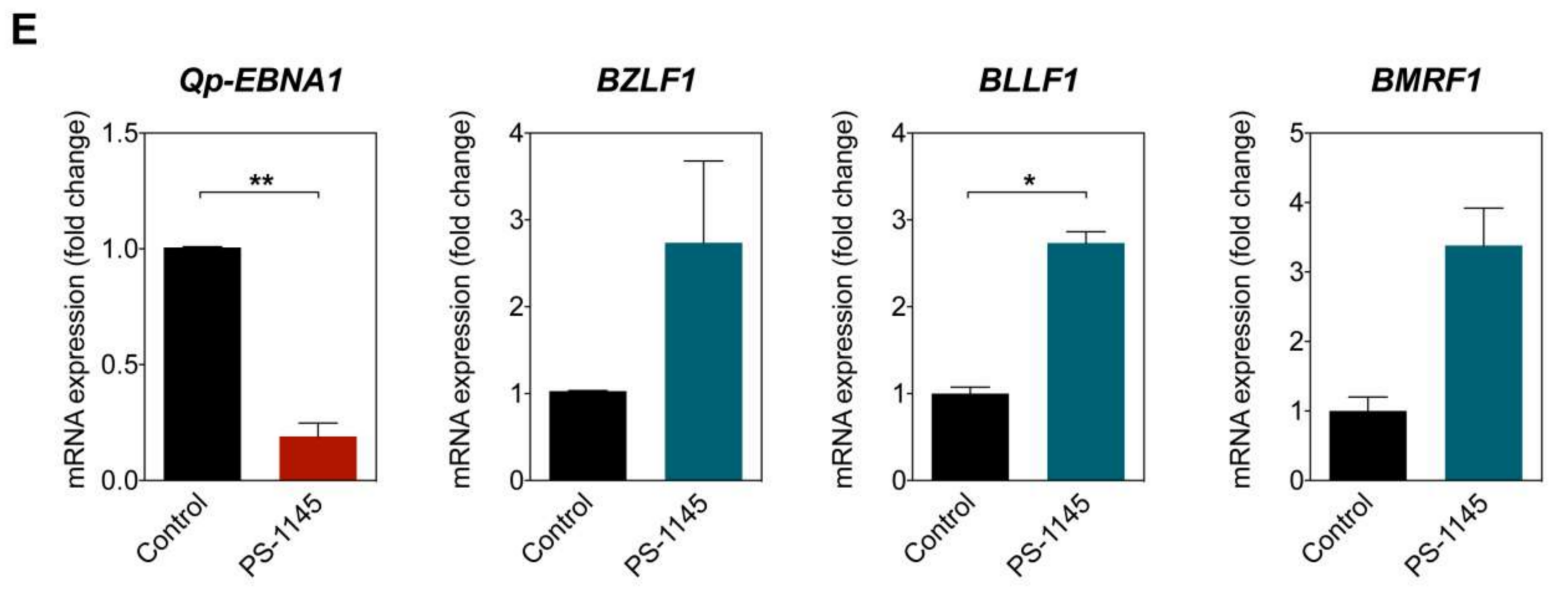
NF-κB p65 activity and *Qp-EBNA1* expression in C666-1 cells. (**A**) Immunofluorescent staining of p65 (green) in C666-1 and HEK 293T cells. Staining of β-tubulin (red) was used as a cytoplasmic control for both cell lines; (**B**) Immunoblot analysis of cytoplasmic and nuclear extracts from C666-1 cells to determine the localization of various transcription factors (p50, p65, Bcl3, STAT3, and IRF2) implicated in the regulation of Qp; (**C**) Schematic showing the inhibition of NF-κB signaling by PS-1145; (**D**) Immunoblot analysis of IκBα phosphorylation to verify the inhibition of NF-κB activity by PS-1145 in C666-1 cells; (**E**) Analysis of the effect of PS-1145 on the expression of *Qp-EBNA1* and of the lytic genes *BZLF1*, *BLLF1*, and *BMRF1* in C666-1 cells by RT-qPCR. Gene expression is shown as fold change mRNA expression relative to that of *GAPDH*. The averages and SEM from three independent experiments are shown. Statistical significance was calculated using the two-tailed Student’s *t*-test. * *p* < 0.05, ** *p* < 0.01.

We then sought to determine the sub-cellular localization of p65 in C666-1, a native EBV-infected NPC cell line, using immunofluorescence microscopy. The HEK 293T cell line, which is not infected with EBV, was used as a negative control. In HEK 293T cells, p65 was clearly expressed exclusively in the cytoplasm (Figure 4A, upper panel). In contrast, the C666-1 cell line displayed both cytoplasmic and nuclear localization of p65 in a substantial number of cells (Figure 4A, lower panel), indicating that the NF-κB pathway is constitutively activated in these cells, resulting in phosphorylation and subsequent nuclear localization of p65. Immunoblot analysis of cellular fractions of C666-1 cells revealed that p50 was present at similar levels in the cytoplasm and nucleus, while only a relatively small fraction of p65 could be detected in the nucleus (Figure 4B). Both Bcl3 and STAT3 were highly expressed in C666-1 cells, but were completely restricted to the cytoplasm, while IRF2 was detectable in the nucleus (Figure 4B). These results demonstrate that both p65 and p50 NF-κB subunits are present in the nucleus of EBV-harboring NPC cell line C666-1.

### 2.6. Inhibition of NF-κB Signaling Downregulates Qp-EBNA1 Expression in C666-1 Cells

To further confirm that *Qp-EBNA1* expression is regulated by NF-κB signaling, we treated C666-1 cells with PS-1145, an inhibitor of IκBα phosphorylation, to inhibit NF-κB activity (Figure 4C) [20]. Immunoblotting showed a significant reduction in phosphorylated IκBα, but not total IκBα or β-tubulin, in C666-1 cells treated with PS-1145, indicating inhibition of NF-κB activity (Figure 4D). We then examined the effect of PS-1145 treatment on *Qp-EBNA1* expression. As expected, inhibition of NF-κB in C666-1 cells resulted in a strong downregulation of *Qp-EBNA1* expression. This result is in line with previous results showing that stimulation of NF-κB signaling induces the expression of *Qp-EBNA1* (Figure 4E). As a reference, the expression of the Zta-encoding immediate-early viral gene *BZLF1*, which is involved in the switch from the latent to the lytic form of EBV infection, and of the lytic genes *BLLF1* and *BMRF1* showed an opposite response following NF-κB inhibition by PS-1145 (Figure 4E). These data indicate that NF-κB plays a vital role in the regulation of *Qp-EBNA1*, as well as in the expression of other EBV latency functions, to maintain EBV latency in EBV-infected NPC cells.

## 3. Discussion

EBNA1 is expressed in all forms of EBV latency in vivo and in EBV-associated tumors because of its crucial roles in viral replication, genome maintenance, and regulation of viral gene expression [24]. As a transcription factor, EBNA1 also regulates cellular genes belonging to pathways dysregulated in oncogenesis. In B cells, EBNA1 has been shown to upregulate CD25, CCL20, RAG1, and RAG2 expression [5,25,26], while, in epithelial cells, EBNA1 induces the expression of STAT1, c-Jun, and ATF2 [27,28]. In EBV-associated malignancies exhibiting latency I or II, such as NPC, EBNA1 expression is driven by Qp; several studies have identified modulators of Qp. We previously found that the JAK/STAT pathway positively regulates Qp activity and that Zta can interfere with JAK/STAT activation, leading to a loss of Qp activity [14]. Our current study demonstrates that NF-κB signaling also plays a role in activating Qp and in the upregulation of *Qp-EBNA1* expression, in addition to positively regulating the *LMP1* and BART promoters. Aberrant NF-κB signaling is a common phenomenon in many malignancies; uncontrolled activation of NF-κB contributes to the initiation and progression of human malignancies by promoting cell survival, transformation, and proliferation, as well as by an immunosuppressive effect [29,30]. Constitutive NF-κB activation has also been reported in NPC and is caused by a combination of genetic abnormalities and upstream factors within the NF-κB pathway and EBV infection [20,31,32]. We demonstrated that stimulation of NF-κB signaling in two immortalized nasopharyngeal epithelial (NPE) cell lines and in the EBV-infected gastric cell line AGS-BX1 resulted in a significant upregulation of *Qp-EBNA1*, *LMP1*, and *RPMS1*. Conversely, treatment of C666-1 cells with the IκB kinase inhibitor PS-1145 inhibited NF-κB activity and downregulated *Qp-EBNA1* expression. The inhibition of NF-κB activity also positively regulated *BZLF1* expression, which indicates that NF-κB plays a role in maintaining EBV latency and preventing its reactivation, as suggested previously.

EBV latency programs are defined by the expression profile of latent genes and the promoters utilized for the expression of *EBNA1*, with Wp/Cp being used for latency III (Wp/Cp) and Qp for latency I/II [1,33]. Wp and Cp are silenced by hypermethylation to evade immune surveillance in EBV latency I and II, while Qp is hypomethylated in all EBV latency programs [34]. Since Qp-EBNA1 latency is found in Burkitt’s lymphoma, nasopharyngeal carcinoma, and EBV-associated gastric carcinoma, it is likely that EBV induces epigenetic silencing of Wp/Cp for tumor immune evasion. Previous studies have proposed a mechanism in which Qp is modulated by a repressor or transactivator, rather than through host epigenetic regulation [9,35]. The presence of the gene for CTCF, a chromatin insulator protein, in the Qp region provides genetic evidence to support the lack of epigenetic silencing of Qp in EBV-infected cells [36]. Therefore, activation of Qp-EBNA1 may rely on the transactivators to displace a Qp-associated repressor and activate the Qp promoter. Besides the NF-κB binding motif described in this study, several transcriptional binding sites, including those recognized by IRF, Sp1, and STAT, have been identified in the Qp promoter region [14,17,37]. Of the other transcription factors known to regulate Qp, IRF2 and IRF7 were found to negatively regulate Qp activity [12,13]. There are also two EBNA1 binding sites immediately downstream of the Qp initiation site, which, when bound by EBNA1, are believed to block Qp activity through a cis-acting mechanism in the setting of the latency III program [6]. Our analysis showed the presence of p65 at varying levels in the nucleus of C666-1 NPC cells, suggesting that p65 is not the only positive regulator that is involved in regulating Qp. Previous studies have characterized a STAT binding site in the Qp region and have suggested that constitutively activated STAT3 signaling in NPC cells may partly contribute to the activation or alleviate repression of Qp [14,37].

**Figure 5:**
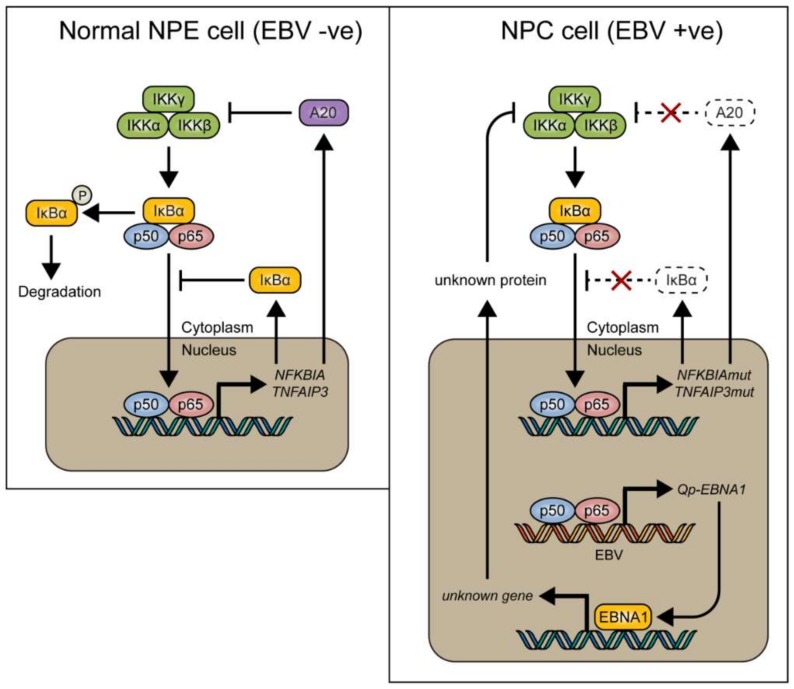
Model showing how NF-κB signaling is tightly regulated by inhibitory proteins such as IκBα and A20 (encoded by the *TNFAIP3* gene) in normal nasopharyngeal epithelial cells (**left**), and how these proteins can be mutated in nasopharyngeal carcinoma (NPC) (**right**), resulting in loss of control of NF-κB signaling. We found that EBV-encoded *Qp-EBNA1* can be upregulated by NF-κB, while EBNA1 protein expression has been shown to negatively regulate NF-κB activation by inhibiting IKKα/β phosphorylation [20]. The regulatory loop between NF-κB signaling and EBNA1 implies an important role for EBV in the control of NF-κB activity in the presence of mutated genes like *NFKBIA* and *TNFAIP3* in NPC.

In conclusion, it is suggested that NF-κB signaling represents one of the positive regulators for Qp expression in NPC cells. However, canonical NF-κB activity must be regulated tightly in NPC cells, since too much activation of NF-κB may induce immune activation or inhibit cell growth. Of note, EBNA1 itself can also inhibit the canonical NF-κB pathway by inhibiting IKKα/β phosphorylation and thereby contribute to cytoplasmic retention of p65 [18,38]. It seems that aberrant signaling causing NF-κB activation, due to host mutations or viral LMP1 activation, may be balanced out by EBNA1 and BART miRNA in EBV-infected NPC cells [20], together with other negative feedback pathways, to make the survival of these cells dependent on the maintenance of a state of EBV latency (Figure 5). Interaction between the EBV latency program and NF-κB signaling in NPC may present a new target for the treatment of EBV-associated NPC.

## 4. Materials and Methods

### 4.1. Cell Lines and Cell Culture Conditions

Human embryonic kidney (HEK) 293T cells were maintained in Dulbecco’s minimal essential medium (DMEM, Gibco, Waltham, MA, USA) supplemented with 10% fetal bovine serum (FBS), 100 units/mL penicillin, and 100 μg/mL streptomycin sulfate (P/S). The EBV-positive nasopharyngeal epithelial (NPE) cell lines NP361hTERT-EBV and NP460hTERT-EBV were grown in a 1:1 mixture of Defined Keratinocyte-SFM (Gibco) and Epilife™ medium (Gibco) with 1% P/S. The EBV-positive NPC cell line C666-1 was grown in RPMI-1640 medium (Gibco) supplemented with 10% FBS and 1% P/S, and the EBV-positive gastric carcinoma cell line AGS-BX1 was cultured in F-12K Nutrient mixture (Gibco) supplemented with 10% FBS and 1% P/S. The cell lines NP460hTERT-EBV and AGS-BX1 were stimulated for 16 h with 0.5 µg/mL poly(I:C) (Invitrogen, Waltham, MA, USA), and NP361hTERT-EBV was stimulated for 16 h with 1 µg/mL poly(I:C) (Invitrogen). C666-1 cells were treated for 48 h with 0.2 mM PS-1145 (Santa Cruz Biotechnology, Dallas, TX, USA) to inhibit NF-κB activity. All cells were cultured at 37 °C with 5% CO_2_.

### 4.2. Plasmid Constructs

The Qp reporter plasmid was constructed by cloning the −100 to +36 Qp DNA sequence described previously into the pGL2 (Amersham Pharmacia, Amersham, Buckinghamshire, UK) luciferase reporter plasmid [39]. The pcDNA3.1-EGFP expression vectors expressing NF-κB p50 and p65 have also been described previously. Mutagenesis of the Qp promoter was performed using the QuikChange site-directed mutagenesis kit (Stratagene, La Jolla, CA, USA) as described by the manufacturer. Primers are listed in Table 1.

### 4.3. ChIP Assay

ChIP assays were performed as described by Nelson et al. (2006) with minor modifications. In short, extracts from C666-1 cells were sonicated using an S-4000 sonicator (Misonix, Farmingdale, NY, USA) and incubated overnight with 5 µg of rabbit anti-p50 (sc-7178, Santa Cruz), 5 µg of rabbit anti-p65 (sc-372, Santa Cruz), or 5 µg of rabbit control IgG (ab46540, Abcam, Cambridge, Cambridgeshire, UK), and antibody–protein–DNA complexes were then pulled-down using Dynabeads Protein A (Invitrogen). The levels of immunoprecipitated DNA were determined by qPCR using a primer pair that amplified the Qp region, including the putative NF-κB site, and a control primer pair (Table 1).

### 4.4. EMSA Analysis

Nuclear extracts for EMSA were prepared as described previously from HEK 293T cells transfected with pcDNA3.1-EGFP-p50 and pcDNA3.1-EGFP-p65. EMSA 5′ IRDye700 oligonucleotides were obtained from Integrated DNA Technologies (IDT) and annealed to obtain double-stranded DNA probes. For the oligo and probe sequences used in the EMSAs, see Figure 2C, lower panel. The binding reactions were performed as described, using the Odyssey Infrared EMSA kit (LI-COR Biosciences, Lincoln, NE, USA). Oligonucleotides in 1300-fold molar excess were used for competition experiments. The super-shift experiments were performed with 2 μg of rabbit anti-p50 (sc-7178, Santa Cruz), rabbit anti-p65 (sc-7151, Santa Cruz), or rabbit control IgG (ab46540, Abcam). The reaction mixtures were run through 5% native polyacrylamide gels for 100 min in 0.5× Tris/Borate/EDTA (TBE) buffer. The gels were scanned immediately with an Odyssey^®^ Infrared Imaging System (LI-COR Biosciences).

### 4.5. Luciferase Reporter Assay

For transfection, HEK 293T cells were seeded at a density of approximately 70% in 24-well plates a day before transfection, with cells being transfected using TransIT^®^-LT1 Transfection reagent (Mirus, Madison, WI, USA). For data normalization purposes, the plasmid phRL-TK (Promega, Madison, WI, USA), expressing Renilla luciferase, was co-transfected with the Firefly reporter plasmid in each experiment. Cell lysates were prepared after 2 days of incubation and examined using a luciferase assay system (Promega), with luciferase activity measured using a Victor3 multilabel plate reader (PerkinElmer, Waltham, MA, USA). All assays were carried out in triplicate, and the results presented are the mean of at least three independent experiments.

### 4.6. Immunoblotting

The cell lysates were fractioned by 10% SDS-PAGE and then blotted onto nitrocellulose membranes (Bio-Rad, Hercules, CA, USA). The membranes were incubated overnight with primary antibodies at a dilution of 1:1000, with the exception of anti-β-tubulin (1:2000). The antibodies used for immunoblotting were: rabbit anti-NF-κB p50 (sc-7178, Santa Cruz), rabbit NF-κB p65 (sc-7151, Santa Cruz), rabbit anti-Bcl-3 (sc-185, Santa Cruz), rabbit anti-STAT3 (sc-482, Santa Cruz), rabbit anti-IRF2 (ab124744, Abcam), rabbit anti-IκBα (9242S, Cell Signaling Technology, Danvers, MA, USA), mouse anti-phospho-IκBα (5A5, Cell Signaling), mouse anti-β-tubulin (T8328, Sigma-Aldrich, St. Louis, MO, USA), and mouse anti-lamin A+C (ab8984, Abcam). The membranes were then incubated with IRDye700-labeled donkey anti-mouse or anti-rabbit, or IRDye800-labeled donkey anti-mouse (LI-COR Biosciences) at a 1:5000 dilution. The blots were detected using an Odyssey^®^ Infrared Imaging System (LI-COR Biosciences).

### 4.7. Quantitative RT-PCR (RT-qPCR)

The RNA was extracted from the cells using RNAiso Plus reagent (TaKaRa, Shimogyō-ku, Kyoto, Japan), and reverse transcription (RT) was performed using random primers with the High-Capacity cDNA Reverse Transcription Kit (Applied Biosystems, Waltham, MA, USA). The qPCR reaction was performed using the SYBR Premix Ex Taq (Tli RNase H Plus) mix (TaKaRa) in a LightCycler 480 instrument (Roche, Penzberg, Upper Bavaria, Germany). The fold change in gene expression was calculated using the comparative crossing point method (2-∆∆CP). Primers and probes used for qPCR are listed in Table 1; gene expression was normalized to that of glyceraldehyde-3-phosphate dehydrogenase (*GAPDH*).

### 4.8. Indirect Immunofluorescence

Microscope coverglasses (Marienfeld GmbH & Co. KG, Lauda-Königshofen, Baden-Württemberg, Germany) with a diameter of 12 mm were coated with 10% poly-l-lysine (Santa Cruz) in water and washed three times with phosphate buffered saline (PBS) in a 24-well plate before cells were seeded onto them. The cells were fixed for 15 min in 4% paraformaldehyde in PBS. The fixated cells were washed two times with PBS and then immersed in 0.5% Triton X-100 in PBS for 3 min to permeabilize the cells, followed by three washes with PBS. The cells were blocked using PBS with 5% normal donkey serum (NDS) overnight at 4 °C, followed by incubation with rabbit anti-p65 (sc372, Santa Cruz) and mouse anti-β-tubulin (T8328, Sigma) at a 1:100 dilution in PBS with 5% NDS for 1 h at 37 °C. After three washes with 0.05% Tween-20 in PBS (PBST), the cells were incubated with secondary antibodies conjugated with different fluorophores at a 1:200 dilution in PBS with 5% NDS for 30 min at room temperature. Finally, the cells were washed three times with PBST, and then the cover glass was transferred onto a small drop of VECTASHIELD antifade mounting medium containing DAPI (Vector Laboratories, Burlingame, CA, USA) on a microscope slide. The cells were examined with a confocal LSM (laser scanning microscope) 710 microscope (Carl Zeiss, Oberkochen, Baden Württemberg, Germany).

### 4.9. Statistics

Statistical analysis was performed using the GraphPad Prism 5 software (GraphPad Software, La Jolla, CA, USA). Significance values (*p* values) were calculated by using a two-tailed Student’s *t*-test with or without a Welch’s correction, depending on whether the samples had equal or unequal variance, as determined by using an F-test.

## 5. Conclusions

There is an auto-regulatory loop for maintenance of EBV latency in NPC cells, in which aberrant NF-κB signaling regulates EBV EBNA1 and BARTs (miR-BARTs and lnc-BARTs) gene expression, while EBNA1 and BARTs in turn keep NF-κB signaling in check for EBV latency program in EBV-infected cells. Therapeutic regimes targeting aberrant NF-κB signaling may provide another option for the treatment of NPC.

## Figures and Tables

**Table 1:** Oligonucleotides used in this study.

Oligo	Sequence (5′–3′)
BCL2L11-F	CAAGAGTTGCGGCGTATTGGAG
BCL2L11-R	ACACCAGGCGGACAATGTAACG
ChIP-Qp-F	GACAGAAATTGGGTGACCACTGAGGG
ChIP-Qp-R	CGCCATCCGGTAGCGCAC
ChIP-Control-F	TCCCTAAGTCACTTTCTTCAAGTTGC
ChIP-Control-R	CGTGCATTTAATTGTGTCTTGTGG
EBV-BLLF1-F	TGTGCTGATAGAGGCTGGTG
EBV-BLLF1-R	TGACACCAAGTCCATCTCCA
EBV-BMRF1-F	AGGAGTGCTGCAGGTAAACC
EBV-BMRF1-R	GCTCTGGTGATTCTGCCACT
EBV-BZLF1-F	AAATTTAAGAGATCCTCGTGTAAAACATC
EBV-BZLF1-R	CGCCTCCTGTTGAAGCAGAT
EBV-LMP1-F	AATTTGCACGGACAGGCATT
EBV-LMP1-R	AAGGCCAAAAGCTGCCAGAT
EBV-Qp-EBNA1-F	GTGCGCTACCGGATGGC
EBV-Qp-EBNA1-R	CATGATTCACACTTAAAGGAGACGG
EBV-RPMS1-F	GAAAAGCTTGGGATTAATGCCTGGACCCTCACCAG
EBV-RPMS1-R	AGGGGATCCCCCGCCACCACGGTGCAGCCTAC
GAPDH-F	GAAGGTGAAGGTCGGAGTA
GAPDH-R	GAAGATGGTGATGGGATTTC
NFKBIA-F	CCCTACACCTTGCCTGTGAG
NFKBIA-R	CGTGTGGCCATTGTAGTTGG
Qp-KpnI-F	CGGGGTACCGACAGAAATTGGGTGACCAC
Qp-HindIII-R	ATCCCAAGCTTCGCCATCCGGTAGCGCAC
Qp-mut1-F	GGTGACCACTGAAAAAGTGTTCCACAG
Qp-mut1-R	CTGTGGAACACTTTTTCAGTGGTCACC
Qp-mut2-F	CTGAGGGAGTGAAAAACAGTAATGTTG
Qp-mut2-R	CAACATTACTGTTTTTCACTCCCTCAG

Underlining indicates altered nucleotides.
